# Genetic testing for indeterminate thyroid cytology: review and meta-analysis

**DOI:** 10.1530/ERC-17-0405

**Published:** 2017-12-18

**Authors:** Sergio Vargas-Salas, José R Martínez, Soledad Urra, José Miguel Domínguez, Natalia Mena, Thomas Uslar, Marcela Lagos, Marcela Henríquez, Hernán E González

**Affiliations:** 1 Department of Surgical OncologyFaculty of Medicine, Pontificia Universidad Católica de Chile , Santiago, Chile; 2 Department of EndocrinologyFaculty of Medicine, Pontificia Universidad Católica de Chile, Santiago, Chile; 3 GeneproDXSantiago, Chile; 4 Department of Internal MedicinePontificia Universidad Católica de Chile, Santiago, Chile; 5 Department of Clinical LaboratoriesFaculty of Medicine, Pontificia Universidad Católica de Chile, Santiago, Chile

**Keywords:** molecular diagnostics, indeterminate thyroid cytology, meta-analysis, accuracy

## Abstract

Thyroid cancer is the most frequent endocrine malignancy, and its incidence is increasing. A current limitation of cytological evaluation of thyroid nodules is that 20–25% are reported as indeterminate. Therefore, an important challenge for clinicians is to determine whether an indeterminate nodule is malignant, and should undergo surgery, or benign, and should be recommended to follow-up. The emergence of precision medicine has offered a valuable solution for this problem, with four tests currently available for the molecular diagnosis of indeterminate cytologies. However, efforts to critically analyze the quality of the accumulated evidence are scarce. This systematic review and meta-analysis is aimed to contribute to a better knowledge about the four available molecular tests, their technical characteristics, clinical performance, and ultimately to help clinicians to make better decisions to provide the best care options possible. For this purpose, we address three critical topics: (i) the proper theoretical accuracy, considering the intended clinical use of the test (rule-in vs rule-out) and the impact on clinical decisions; (ii) the quality of the evidence reported for each test (iii) and how accurate and effective have the tests proved to be after their clinical use. Together with the upcoming evidence, this work provides significant and useful information for healthcare system decision-makers to consider the use of molecular testing as a public health need, avoiding unnecessary surgical risks and costs.

## Introduction

Thyroid cancer is the most frequent endocrine malignancy, and its incidence is rapidly increasing (Pellegriti *et al.* 2013). In fact, some studies have projected that the number of patients diagnosed with thyroid cancer in the US could rise from 56,000 cases in 2017 to 183,000 cases in 2030 (Rahib *et al.* 2014). This is most likely due to the expanded availability of thyroid ultrasound and subsequent increment of diagnostic fine-needle aspiration biopsies (Sosa *et al.* 2013, Davies & Welch 2014). A current limitation of cytological evaluation is that approximately 20–25% will be reported as indeterminate, i.e. Bethesda III (atypia of undetermined significance or follicular lesion of undetermined significance) and Bethesda IV (follicular neoplasm or suspicious for a follicular neoplasm) (Cibas & Ali 2009, Faquin *et al.* 2011).

The challenge of indeterminate thyroid cytology (ITC) is to determine whether the nodule is malignant and should undergo surgery, or benign, and should be recommended to follow-up (Haugen *et al.* 2016). Until 2009, a significant number of patients with ITC underwent diagnostic surgery based on a malignancy probability ranging between 15 and 40% (Haugen *et al.* 2016). However, the emergence of precision medicine has changed the paradigm of ITC management. The first test released to the market was the Afirma gene expression classifier (Afirma-GEC) (Alexander *et al.* 2012), followed by ThyGenX/ThyraMIR (Labourier *et al.* 2015), ThyroSeq v2 (Nikiforov *et al.* 2014) and RosettaGX Reveal (Lithwick-Yanai *et al.* 2017). These tests claim to improve the preoperative diagnosis of nodules ITC nodules, potentially avoiding thousands of unnecessary surgeries (Nishino 2015) and in some cases to guide the surgical approach (Shrestha *et al.* 2015). Since these novel tests have become available for clinical use, evidence reporting clinical experiences has accumulated. However, efforts to critically analyze the quality of the evidence are scarce (e.g. Nishino 2015).

Precision medicine is usually understood as ‘the right treatment, for the right patient, at the right time’ (Mirnezami *et al.* 2012). Similarly, ‘the right clinical decision, for the right cytology, with the right test’ should be considered to perform an appropriate molecular diagnosis for ITC. Therefore, a comprehensive analysis of the available evidence supporting different tests will provide clinicians with a better understanding of tests performance and interpretation, while helping to appropriately select patients that will benefit from molecular testing. This review is aimed to contribute to a better knowledge of the available molecular tests, their technical characteristics and clinical performance, through a critical and systematic review and a meta-analysis of the current literature reporting results for the four commercially available tests for ITC cytology.

## Previous considerations about molecular diagnosis of indeterminate thyroid cytology

### Should the tests rule-in or rule-out?

One of the most important challenges in binary classification tests is to choose the proper limits of the clinical sensitivity and specificity, in order to minimize the misdiagnosis (Lalkhen & McCluskey 2008). Optimal values for sensitivity and specificity depend on the intended use of a laboratory test. Ultimately, physicians drive their decisions by estimating the disease probability in the specific scenario of a positive or a negative test result. This information is summarized in the positive predictive value (PPV, the probability of presenting the disease when the test is positive) and the negative predictive value (NPV, the probability of being free of disease when the test is negative) (Altman & Bland 1994). According to the Bayes Theorem (Hall 1967), predictive values follow a function that mathematically connect the sensitivity and specificity of a test with the disease prevalence. Thus, changes in sensitivity and specificity will directly affect PPV and NPV.

Applied to the clinical scenario of ITC, when the diagnostic test is intended to predict benign nodules, it will require a high NPV, while to predict malignancy, it will need to have a high PPV. In the following section, we elaborate on these concepts and discuss in detail the statistical parameters that need to be considered for ITC diagnostic tests and what diagnostic performance would be required to be clinically effective.

#### Rule-out tests

**Figure 1:**
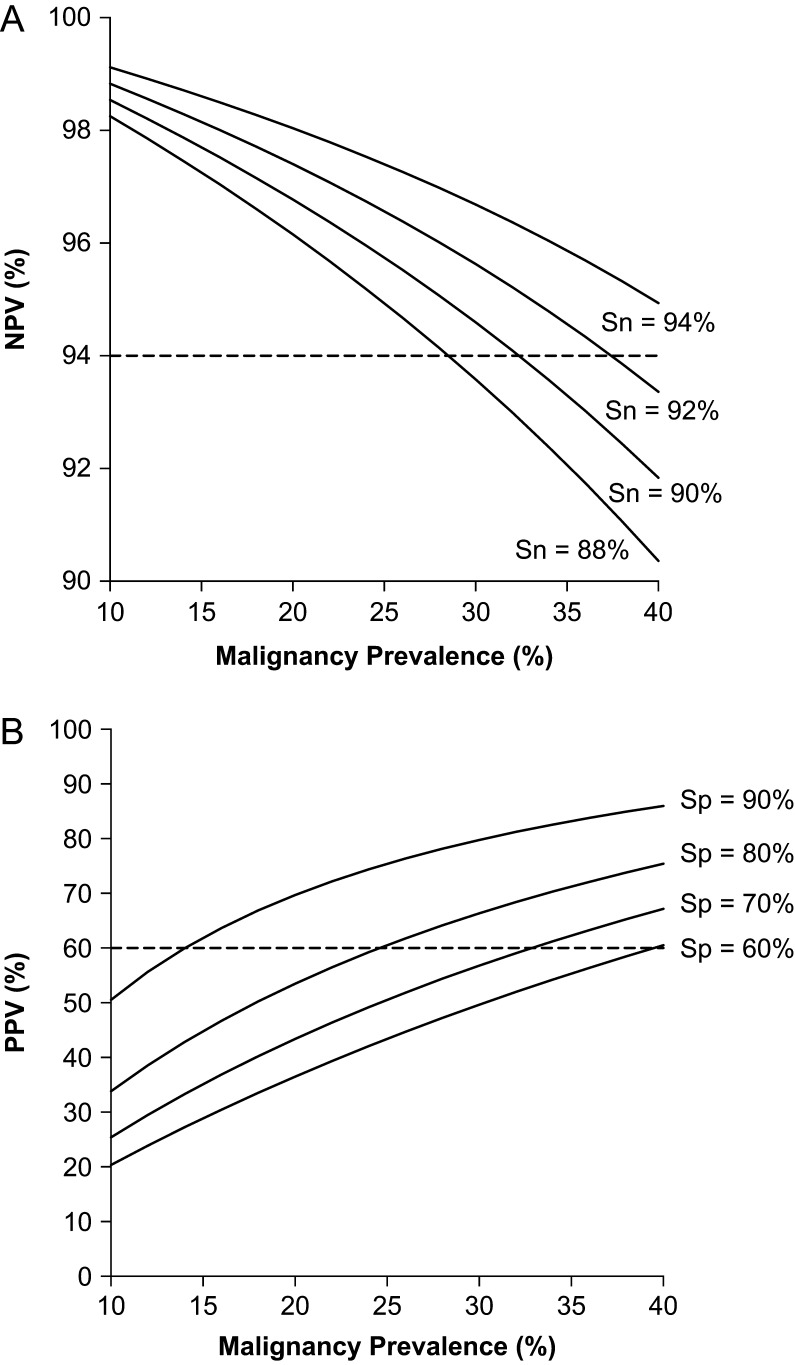
Simulations of the negative predictive value (NPV) (A) and of the positive predictive value (PPV) (B) as functions of the malignancy prevalence in indeterminate thyroid cytology. For NPV (A), specificity was fixed at 75% and four curves were simulated at sensitivities between 88% and 94%. For PPV (B), sensitivity was fixed at 92% and four curves were simulated at specificities between 60% and 90%. Dashed lines highlight the threshold of minimum theoretical NPV and PPV values considered as limits for clinically useful tests.

Since patients with Bethesda III and IV cytology are frequently recommended to undergo diagnostic surgery (which is unnecessary in approximately in 75% of cases) (Haugen *et al.* 2016), the first relevant clinical question is whether a patient could be followed up or not. This question is answered by rule-out tests aimed to predict benign thyroid nodules. Statistically, the test should have a NPV of at least 94% with a residual risk of malignancy lower than 6% for a negative result, closer to a Bethesda II cytology (Cibas & Ali 2009). Basically, this component will determine how safe the test will be when clinical follow-up is recommended instead of diagnostic surgery. Since the NPV depends on disease prevalence, it is necessary to determine a minimum sensitivity that will keep the NPV above 94% in a broad range of disease prevalence. In Fig. 1A, the NPV was simulated at different sensitivities, showing that sensitivity above 90% would result in a NPV above 94% in disease prevalence below 35%.

Another informative parameter to consider when evaluating a rule-out test is the specificity. In this clinical context, the specificity of the test informs about the proportion of benign nodules effectively detected by the test. In other words, the specificity of the test will determine how many patients with benign nodules will be correctly identified and will ultimately avoid diagnostic surgery. For example, if a test has a specificity of 50% (with a cancer prevalence of 25%), in 100 patients with ITC approximately 38 of 75 benign cases will avoid surgery. When test specificity rises to 90%, 68 of the 75 patients with benign nodules would be able to avoid surgery. Clearly, a higher number of patients will benefit with the second test and, assuming the same cost for both tests, it is likely that there will be greater economical savings as well. Therefore, in a rule-out test, the specificity directly impacts in the cost-effectiveness.

#### Rule-in tests

When the intended use of the test is to predict malignancy, it is called a rule-in test (Deeks & Altman 2004), and it will drive the clinician to recommend surgery in case of a positive result. From a clinical point of view, there is no agreement on which is the minimum PPV required to change the clinical decision toward recommending surgical treatment. Since the standard of care in ITC patients is frequently surgery, a minimal post-test probability for malignancy of 50–75%, closer to a Bethesda V cytology (Cibas & Ali 2009), could be considered appropriate for a rule-in test. In Fig. 1B, the PPV was simulated at different specificities, showing that specificity above 80% would result in a PPV above 60% in disease prevalence above 25%. Some authors have suggested that rule-in tests could also help to guide surgery (i.e. thyroid lobectomy vs total thyroidectomy), given that most of these tests identify high-risk mutations associated with poorer outcomes (Shrestha *et al.* 2015, Kargi *et al.* 2016). The Cancer Genomic Atlas (TCGA) project has shown very clarifying data allowing us to further stratify thyroid cancer patients (Cancer Genome Atlas Research Network 2014, Asa *et al.* 2015), based on a BRAF-like or RAS-like profile and potentially provides actionable information for surgeons to tailor the surgical approach. However, although it seems reasonable to be more surgically aggressive in patients with a high-risk mutational profile, there is no evidence that changing the surgical approach improves oncological outcomes. Furthermore, defining the optimal surgical approach is an entirely different clinical question, which needs to consider the risk of clinically relevant recurrent structural disease, rather than the risk of malignancy. Therefore, further studies will be needed to determine if ITC patients with both, malignant result by a rule-in test and high-risk mutational profile, could benefit from more aggressive surgical approaches.

#### Impact of disease prevalence

Since the PPV and NPV of a test are a function of both, the disease prevalence and the test accuracy (sensitivity and specificity) (Altman & Bland 1994), it is important to understand how predictive values (i.e. the post-test probability) change with variations in the malignancy pre-test probability. Based on the Bayes’ Theorem, Fig. 1 shows a simulation analysis of NPV (Fig. 1A) and PPV (Fig. 1B) as a function of the malignancy prevalence. Note that, for a malignancy prevalence between 20 and 40%, the NPV remains above 94% (accepted limit for rule-out tests) for sensitivities above 90%. In a prevalence of 20–40%, a specificity above 80% is required for a PPV of 60% (accepted limit for rule-in tests). Therefore, considering a cancer prevalence range of 20–40%, a specificity of 80% or more could be considered as an optimal standard for ruling-in, while the minimum sensitivity for a robust rule-out test would be 90%.

### What sensitivity/specificity combination is required for a truly effective test?

**Figure 2:**
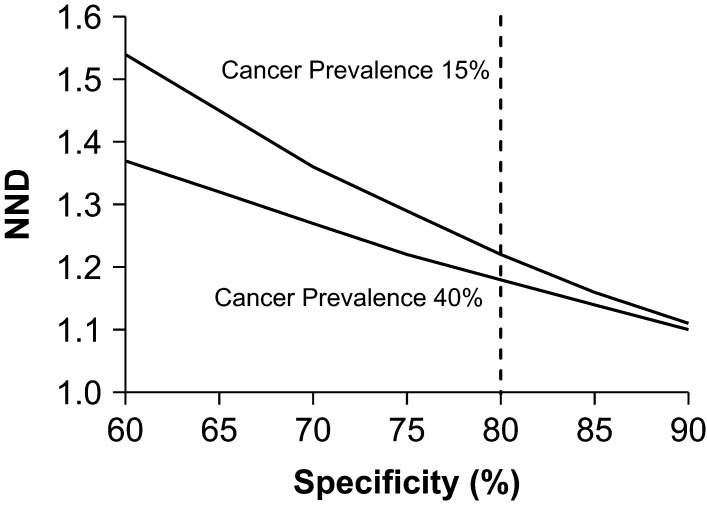
Simulation of the number of tests needed to correctly diagnose one indeterminate thyroid cytology (NND) at different specificities, considering a cancer prevalence of 15% and 40%. Dashed line indicates a specificity of 80%.

From a clinical point of view, we have shown that a sensitivity of 90% and specificity of 80% are good parameters to determine if a test for ITC will perform adequately in a broad range of disease prevalence. Nevertheless, another important parameter to be considered is the balance between the clinical accuracy (i.e. the impact on individual patients) and the clinical effectiveness (i.e. the impact on the whole population). Before molecular testing, a significant number of ITC patients underwent diagnostic surgery, but only 15–40% of those patients were diagnosed with malignant nodules. Assuming that benign nodules were ‘unnecessarily’ diagnosed by surgery (since their treatment should be not surgical), the number of surgeries needed to diagnose one malignant case ranges from 2.5 to 7. However, considering a sensitivity of 90% and specificity of 60–90%, the number of tests needed to correctly diagnose a thyroid nodule is consistently lower than 2.5. Figure 2 shows a simulation of the number needed to diagnose (NND) based on the aforementioned values of sensitivity and specificity. Interestingly, when specificity is 80% or more, the NND is similar in the whole range of cancer prevalence (15–40%). Therefore, from a clinical-effectiveness point of view, the same specificity considered for a proper clinical accuracy should be enough to obtain an effective test in a broad population. Taken together, this analysis suggests that a sensitivity of 90% and specificity of 80% are required for an ideal molecular test for ITC, given that these parameters are associated to both, optimal clinical accuracy and clinical effectiveness.

### Other clinical considerations

When considering molecular testing for ITC, the clinician should carefully consider the intended use of the test (rule-in or rule-out) to determine if results will truly impact the clinical decision, Thus, other clinical factors should be considered, such as patient preferences, risk of malignancy based on ultrasound features and the therapeutic options that are being considered for a specific case. Before testing, patients should clearly understand how the test may change the risk of malignancy and the potential clinical recommendations based on testing results. Also, if ultrasound features of an ITC are of high risk (microcalcifications, irregular borders and hypoechoic), the thyroid nodule is large or there is a multinodular goiter, and the clinician should be clear if the test result will change the clinical recommendation, since these factors may have greater weight in driving the clinical decision, thereby reducing the utility of the test. Therefore, it seems reasonable to consider the ultrasonographic characteristics as a primary filter to select the most appropriate nodule to test. In cases of more than one ITC, the clinician should evaluate if a benign test result will suffice to recommend watchful waiting and avoid surgery. Finally, since the intended use of molecular tests is to determine the probability of malignancy and there are false-negative results, caution should be taken if results will be used to choose non-surgical options such as thermoablation.

## Systematic review of the evidence

### Methods

#### Literature searching and systematic review

This review was performed by following the guideline for Medical Test Review from the Agency for Healthcare Research and Quality (AHRQ) and the Evidence-based Practice Centers (EPC) (AHRQ 2017). Three reviewers independently searched MEDLINE/PubMed (NIH interface), EMBASE, Google Scholar and Epistemonikos to identify potentially relevant articles or abstracts. The key words were as following: diagnosis OR ‘gold standard’ OR ‘ROC’ OR ‘receiver operating characteristic’ OR sensitivity OR specificity OR likelihood OR ‘false positive’ OR ‘false negative’ OR ‘true positive’ OR ‘true negative’ OR ‘predictive value’ AND ‘indeterminate’ AND ‘thyroid nodules’ AND ‘molecular’. The search included all the results delivered up to May 30, 2017. There were no language restrictions. For the final review and meta-analysis, three reviewers independently screened studies and determined the study eligibility. The inclusion criteria were primary studies, measurement of diagnostic accuracy parameters and use of clinically validated tests. The critical evaluation of the studies was performed by following a standardized checklist summarizing the AHRQ/EPC guideline (Supplementary material, see section on supplementary data given at the end of this article) (AHRQ 2017). Disagreements were resolved by consensus of the three reviewers. The primary outcomes considered for analysis were sensitivity and specificity.

#### Meta-analysis

Only studies reporting results on pathologically validated samples (i.e. with surgical biopsy report available) were considered for meta-analysis. The primary statistical analysis was based on the Rutter-Gatsonis hierarchical summary receiving operator characteristic (HSROC) model (Rutter & Gatsonis 2001) as suggested by the AHRQ/EPC guideline (AHRQ 2017), using the software RStudio. The *I* ^2^ statistic was calculated to determine the proportion of between-study variation by heterogeneity, with suggested thresholds for very low (0–24%), low (25–49%), moderate (50–74%), and high (75%) values (Higgins & Thompson 2002). Accuracy was assessed by calculating the logarithmic diagnostic odd ratio (log-DOR), i.e. the ratio between positive LR and negative LR. When the log-DOR is greater than zero, and the confidence interval does not cross the line of nullity, it favors the use of the test (Glas *et al.* 2003). For studies in which specificity could not be calculated, a theoretical negative LR was considered based on the specificity reported in the clinical validation. For studies with repeated measurements, a relevant-time correction was performed as suggested by Peters & Mengersen (Peters & Mengersen 2008).

### Results

#### General description

**Figure 3:**
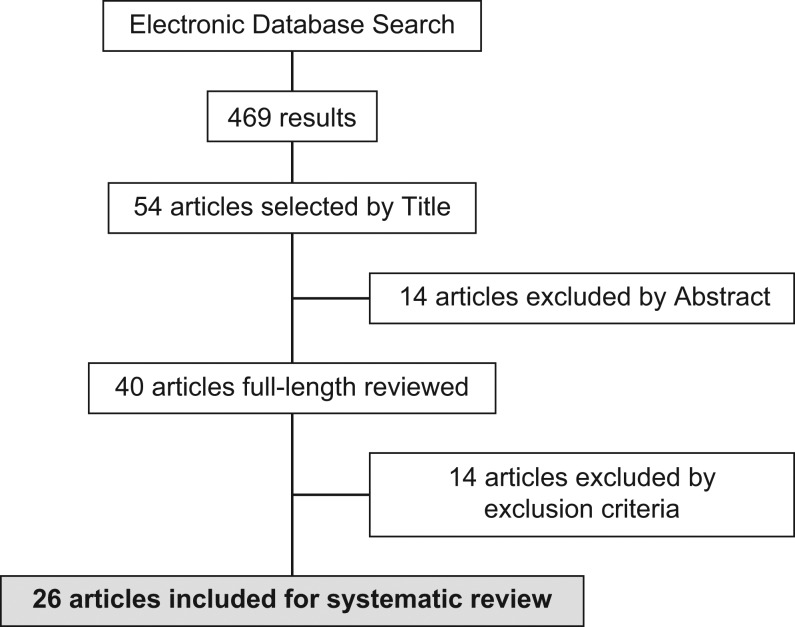
Flowchart of evidence searching.

The electronic database search delivered 469 results, from which 54 article titles were chosen by three independent reviewers. These articles included original research, reviews and short articles. The 54 respective abstracts were analyzed and 40 articles meeting inclusion criteria were selected for full-length reading. Twenty-seven studies were selected for cross-matching between reviewers. The Cohen-kappa statistic (Viera & Garrett 2005) for reviewer’s agreement was 0.95, and one study was excluded (Angell *et al.* 2015) given the lack of consensus, mainly based on a low sample size (*n* = 5) and absence of gold standard for samples called ‘benign’. Finally, 26 studies were included for systematic review: 19 studies for Afirma-GEC, five studies for ThyroSeq v2, one study for ThyGenX/ThyraMIR and one study for RosettaGX Reveal (Fig. 3). For Afirma-GEC, one study was performed in a clinical validation stage (Alexander *et al.* 2012) and 18 in a post-validation stage (Alexander *et al.* 2014, Harrell & Bimston 2014, Lastra *et al.* 2014, McIver *et al.* 2014, Sullivan *et al.* 2014, Celik *et al.* 2015, Marti *et al.* 2015, Witt 2015, Yang *et al.* 2015, Zhu *et al.* 2015, Chaudhary & Li 2016, Samulski *et al.* 2016, Wu *et al.* 2016, Al-Qurayshi *et al.* 2017, Baca *et al.* 2017, Harrison *et al.* 2017, Kay-Rivest *et al.* 2017, Roychoudhury *et al.* 2017). One post-validation study was performed outside of the US. Among the post-validation studies, five were multicentric and 13 were single-center (Table 1). For ThyroSeq v2, two studies were performed in a clinical validation stage (Nikiforov *et al.* 2014, 2015), and three were post-validation experiences (Shrestha *et al.* 2016, Toraldo *et al.* 2016, Valderrabano *et al.* 2017). All the studies were performed in the US, in a single-center setting (Table 1). Although there were some post-validation studies that individually analyzed ThyGenX (8-gene mutational panel), no post-validation studies were identified for the current combined version ThyGenX/ThyraMIR, nor the recently released RosettaGX Reveal test. Thus, only the multicentric clinical validation studies were included (Labourier *et al.* 2015, Lithwick-Yanai *et al.* 2017) (Table 1).

**Table 1:** Body-of-evidence summary.

First author	Year	Patients analyzed	Bethesda-FNA setting	Test	Test-development stage	Country	Centres included	Evidence quality	Body-of-evidence quality
Alexander	2012	210	III–IV	Afirma-GEC	Clinical validation	US	Multicentric	High	High
Harrel	2013	35	III–IV	Afirma-GEC	Post-validation	US	1 center, Florida	High	
Alexander	2014	132	III–IV	Afirma-GEC	Post-validation	US	Multicentric	High	
McIver	2014	36	III–IV	Afirma-GEC	Post-validation	US	Multicentric	High	
Sullivan	2014	7	III–IV	Afirma-GEC	Post-validation	US	1 center, Los Angeles	Moderate	
Lastra	2014	50	III–IV	Afirma-GEC	Post-validation	US	1 center, Philadelphia	High	
Celik	2015	32	III–IV	Afirma-GEC	Post-validation	US	1 center, Florida	High	
Marti	2015	70	III–IV	Afirma-GEC	Post-validation	US	Multicentric	Moderate	
Witt	2015	15	III–IV	Afirma-GEC	Post-validation	US	1 center, Newark	Moderate	
Yang	2015	67	III–IV	Afirma-GEC	Post-validation	US	1 center, Los Angeles	High	
Zhu	2015	10	III–IV	Afirma-GEC	Post-validation	US	1 center, Boston	Poor	
Al-Qurayshi	2016	112	III–IV	Afirma-GEC	Post-validation	US	Multicentric	High	
Chaudhary	2016	81	III–IV	Afirma-GEC	Post-validation	US	1 center, Columbus	High	
Samulski	2016	123	III–IV	Afirma-GEC	Post-validation	US	1 center, Philadelphia	High	
Wu	2016	95	III–IV	Afirma-GEC	Post-validation	US	1 center, Los Angeles	Moderate	
Baca	2017	110	III–IV	Afirma-GEC	Post-validation	US	1 center, Boston	High	
Harrison	2017	49	III–IV	Afirma-GEC	Post-validation	US	1 center, N. Carolina	Moderate	
Roychoudhury	2017	60	III–IV	Afirma-GEC	Post-validation	US	Multicentric	High	
Kay-Rivest	2017	77	III–IV	Afirma-GEC	Post-validation	Canada	1 center, Montreal	Moderate	
Nikiforov	2014	52	IV	ThyroSeq v2	Clinical validation	US	1 center, Pennsylvania	Moderate	Intermediate
Nikiforov	2015	95	III	ThyroSeq v2	Clinical validation	US	1 center, Pennsylvania	High	
Shrestha	2016	56	III–IV	ThyroSeq v2	Post-validation	US	1 center, Minneapolis	Moderate	
Toraldo	2016	45	III–IV	ThyroSeq v2	Post-validation	US	1 center, Boston	Poor	
Valderrabano	2017	102	III–IV	ThyroSeq v2	Post-validation	US	1 center, Florida	High	
Labourier	2015	109	III–IV	ThyGenX/ThyraMIR	Clinical Validation	US	Multicentric	Moderate	Low
Lithwick-Yanai	2017	150	III–IV	RosettaGX Reveal	Clinical Validation	US–Eur–Israel	Multicentric	Moderate	Low

#### Evidence quality assessment

##### Afirma-GEC

Originally, all studies were designed to examine the diagnostic accuracy of Afirma-GEC with clearly defined eligibility criteria for participants (non-bias evidence). For the clinical validation study, patients were prospectively enrolled. Four post-validation studies included prospective cohorts, while 14 studies analyzed retrospectively enrolled patients. For primary analysis of the diagnostic accuracy, the clinical validation study provided a sample size estimation based on proper statistical methods, while the remaining studies used a convenience sample size. Importantly, the clinical validation study was double-blinded, i.e. the test result was unknown when the gold standard was reported, and the gold standard was unknown when the test result was interpreted.

For primary clinical validation, the surgical biopsy report was considered as the gold standard. However, for most of the post-validation studies, not all the gold standards were available since the Afirma-GEC results have been considered for clinical decisions, so most of the ‘benign’ cells were recommended follow-up by assumption that these cases were truly benign. Therefore, only some post-validation analyses are based on cases with surgical pathology gold standard, including Afirma-GEC called ‘suspicious’ and a few ‘benign’ cases, which ultimately underwent surgery. Four studies did not report a gold standard for Afirma-GEC ‘benign’ results, although they provided information about how many nodules were diagnosed as benign (Witt 2015, Zhu *et al.* 2015, Kay-Rivest *et al.* 2017, Roychoudhury *et al.* 2017). Only one study (Witt 2015) reported a 426-day follow-up for patients called ‘benign’, with no evidence of a false-negative result. Regarding the sources of heterogeneity, no statistically significant differences were found, but a trend to a poorer overall performance when the sample size was smaller or when the gold standard was not available for Afirma-GEC called ‘benign’ was observed.

In summary, twelve studies (including the clinical validation) showed high evidence quality, six showed moderate quality and one showed poor quality (Table 1). Considering the number of post-validation studies (including 1161 patients with gold standard), with high evidence quality in most of them, the body of evidence for Afirma-GEC was graded as high. Further details about the quality assessment of the primary clinical validation are described in Table 2.

**Table 2:** Quality assessment for clinical validation studies

	Afirma-GEC	ThyroSeq v2	ThyGenX/ThyraMIR	RosettaGX Reveal
Methodology	mRNA microarray for 167 genes	Next-generation sequencing for 56 genes	Multiplex PCR for 8-gene genetic alterations and 10 miRNA expression	qPCR profile for 24 miRNA
Adequacy of reporting	Good	Good	Moderate	Poor
Samples are prospectively collected	Yes	Partially	Yes	Yes
Inclusion/exclusion criteria are clearly specified	Yes	Yes	Yes	Yes
Sample size is calculated by statistical methods	Yes	Yes	No	No
Validation cohort represents the target population	Partially (Bethesda V included)	Yes	Yes	Partially (Bethesda II, V and VI included)
Surgical biopsy report is considered as the gold standard	Yes	Yes	Yes	Yes
Surgical biopsy report is unknown when the test is interpreted	Yes	Yes	Not clear	Yes
Test result is unknown when the surgical biopsy report is delivered	Yes	No	Not clear	Yes
Diagnostic accuracy is properly analyzed (including a 2 × 2 cross-tabulation)	Yes	Yes	Not separated by Bethesda III and IV	No
Ethics issues are properly addressed	Yes	Yes	Yes	Yes

##### ThyroSeq v2

All studies for ThyroSeq v2 properly assessed the diagnostic accuracy by following standardized inclusion criteria in a statistically significant sample size. Separate clinical validation studies for ThyroSeq v2 were performed in cases with Bethesda III (Nikiforov *et al.* 2015) and Bethesda IV (Nikiforov *et al.* 2014) cytology. For the Bethesda III study, the cohort was prospectively collected, while a significant number of patients (64%) were included retrospectively in the Bethesda IV study. The largest post-validation study included 102 patients in one non-sponsored center, with a low overall risk of bias and high evidence quality. Additionally, two independent studies with smaller cohorts have been reported, with poor-moderate overall quality (Table 1). Primary clinical validation studies met all the general requirements for an adequate and good quality reporting, including a large representative sample size, and mitigated risk of bias when recruiting and analyzing the diagnostic accuracy, but only single-blinded (Table 2).

##### ThyGenX/ThyraMIR and RosettaGX Reveal

Both, ThyGenX/ThyraMIR and RosettaGX Reveal tests have reported multicentric clinical validation studies (RosettaGX Reveal included samples from European and Israeli cohorts) (Table 1). In both cases, samples were prospectively collected with clearly specified inclusion/exclusion criteria; however, details about the sample size calculation were not reported (Table 2). Importantly, when the representativeness of the cohorts and the accuracy analysis were assessed, we identified a potential source of bias for the RosettaGX Reveal clinical validation study. Specifically, samples in which there was no agreement on the gold standard were excluded; interestingly, all the 17 malignant samples excluded (of 31 cases) were misclassified. This represents a source of differential verification bias that should be avoided. For ThyGenX/ThyraMIR, the double-blinding was not clearly specified in the clinical validation study report, and there was no cross-tabulated diagnostic analysis available for individual Bethesda III and Bethesda IV setting of samples (Table 2). Given that there is no accumulated post-validation evidence available for both tests, the overall grade of the body of evidence is low compared with Afirma-GEC and ThyroSeq v2 (Table 1).

#### Diagnostic accuracy from clinical validation studies

##### Bethesda III and Bethesda IV composition

The four clinical validation cohorts showed interesting differences in their composition. The proportion of Bethesda III cytology was 61% for Afirma-GEC and 65% for ThyroSeq v2, while Bethesda IV cytology was 39% and 35%, respectively. Cancer prevalence was approximately 25% for both, Bethesda III and IV (Table 3). ThyGenX/ThyraMIR cohort showed a slightly different composition. Bethesda III and IV showed a 1:1 ratio (52% and 48% respectively), and the cancer prevalence was 32–33% (Table 3). The RosettaGX Reveal cohort was predominantly composed by Bethesda IV cytologies (87%), with an overall cancer prevalence of 21% on ITC (Table 3), while information about the specific cancer prevalence in Bethesda III and Bethesda IV was not available.

**Table 3:** Paired sensitivity, specificity, negative predictive value (NPV) and positive predictive value (PPV) of clinical validation studies.

Test	Bethesda	Patients analyzed	TP	FN	TN	FP	Cancer prevalence (%)	Sensitivity (%; 95% CI)	Specificity (%; 95% CI)	NPV (%; 95% CI)	PPV (%; 95% CI)
Afirma-GEC	III–IV	210	46	5	82	77	24	90 (78–96)	52 (44–60)	94 (86–98)	37 (29–47)
ThyroSeq v2	III–IV	147	32	4	102	9	24	89 (73–96)	92 (85–96)	96 (90–99)	78 (62–89)
ThyGenX/ThyraMIR	III–IV	109	31	4	63	11	32	89 (72–96)	85 (75–92)	94 (85–98)	74 (58–86)
RosettaGX Reveal	III–IV	150	23	8	88	31	21	74 (55–87)	74 (65–81)	92 (84–96)	43 (30–57)
Afirma-GEC	III	129	28	3	52	46	24	90 (73–97)	53 (43–63)	95 (84–99)	38 (27–50)
ThyroSeq v2	III	95	20	2	67	6	23	91 (69–98)	92 (82–97)	97 (89–99)	77 (56–90)
ThyGenX/ThyraMIR	III	57	17	1	31	8	32	94 (71–99)	79 (63–90)	97 (82–99)	68 (46–84)
RosettaGX Reveal	III	29	Information not available								
Afirma-GEC	IV	81	18	2	30	31	25	90 (67–98)	49 (36–62)	94 (78–99)	37 (24–52)
ThyroSeq v2	IV	52	12	2	35	3	27	86 (56–97)	92 (78–98)	95 (80–99)	80 (51–95)
ThyGenX/ThyraMIR	IV	52	14	3	32	3	33	82 (56–95)	91 (76–98)	91 (76–98)	82 (56–95)
RosettaGX Reveal	IV	131	Information not available								

CI, confidence interval; FN, false negatives; FP, false positives; NPV, negative predictive value; PPV, positive predictive value; TN, true negatives; TP, true positives.

##### Overall diagnostic accuracy

Sensitivity was consistently close to 90% with similar 95% confidence interval for Afirma-GEC, ThyroSeq v2 and ThyGenX/ThyraMIR. RosettaGX Reveal showed a sensitivity of 74% when considering the whole cohort, and 100% when non-agreement gold standard cases were excluded. Specificity was similar for ThyroSeq v2 and ThyGenX/ThyraMIR (92% and 85% respectively, without statistically significant difference). These tests showed higher specificities than RosettaGX Reveal (74%) and Afirma-GEC (52%) (Table 3). No statistical differences were found for the NPV between the four tests, ranging between 92% and 96%. However, ThyroSeq v2 and ThyGenX/ThyraMIR showed a comparable PPV of 74–78%, which was significantly higher than the PPV reported by both, Afirma-GEC and RosettaGX Reveal (37 and 43% respectively) (Table 3).

##### Specific diagnostic accuracy for Bethesda III and Bethesda IV

The sub-analysis of diagnostic accuracy for Bethesda III and Bethesda IV cytologies was performed considering Afirma-GEC, ThyroSeq v2 and ThyGenX/ThyraMIR. RosettaGX Reveal was excluded since they did not provide information about the diagnostic performance in these specific categories. For Bethesda III cytology, no differences were observed for sensitivity between Afirma-GEC, ThyroSeq v2 and ThyGenX/ThyraMIR (Table 3). For Bethesda IV, the ThyGenX/ThyraMIR study showed a non-significant trend to a lower sensitivity when compared to Bethesda III samples (82% vs 94%, respectively) (Table 3). Both, Afirma-GEC and ThyroSeq v2 specificities remained invariable for Bethesda III (90% for Afirma-GEC, 91% for ThyroSeq v2) and IV (90% for Afirma-GEC, 86% for ThyroSeq v2), but was slightly higher for Bethesda IV than Bethesda III cytologies in case of ThyGenX/ThyraMIR (91% for Bethesda IV, 79% for Bethesda III). ThyroSeq v2 and ThyGenX/ThyraMIR showed higher specificity than Afirma-GEC for both, Bethesda III and Bethesda IV samples (Table 3). For all the three tests, the NPV for Bethesda III and IV was at least 91%, with no differences between them. Alternatively, the PPV was significantly higher in ThyroSeq v2 and ThyGenX/ThyraMIR when compared to the Afirma-GEC (37–38%), showing a slightly higher performance in Bethesda IV (80–82%) with respect to Bethesda III samples (68–77%) (Table 3).

#### Meta-regression of diagnostic accuracy

**Figure 4:**
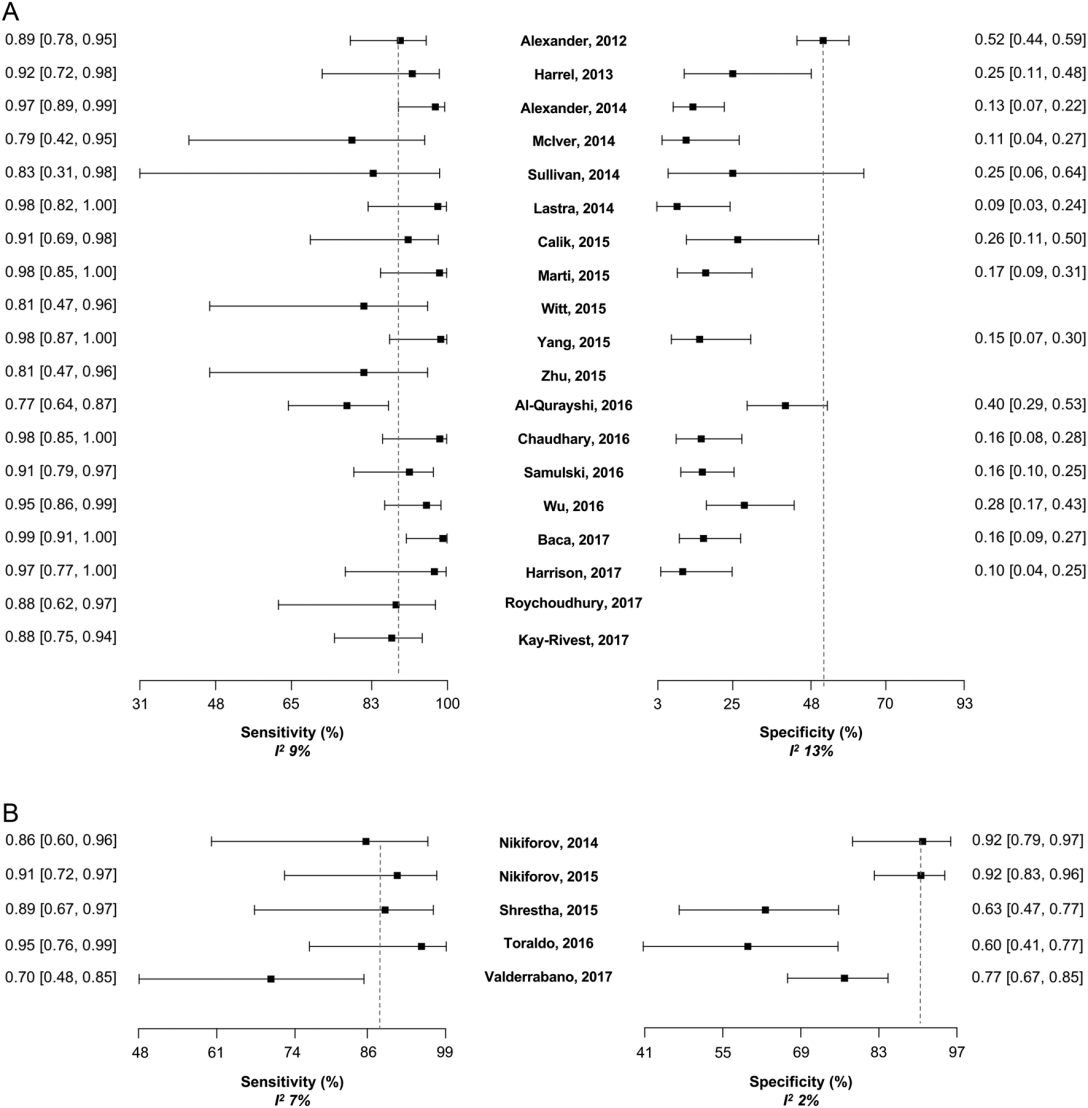
(A) Forest plot of sensitivities and specificities reported in the 19 studies of Afirma-GEC. For each one, the dot represents the absolute value of sensitivity or specificity, and lines represent the 95% of confidence interval. The specificity was not available in four studies, since there was no gold standard for benign cases. The values of sensitivity (with 95% of confidence interval) appear at the left of the figure and at the right for the specificity. (B) Forest plot of sensitivities and specificities reported in the 5 studies of ThyroSeq, v2. Dashed lines indicate the sensitivity and specificity of original validation studies.

**Figure 5:**
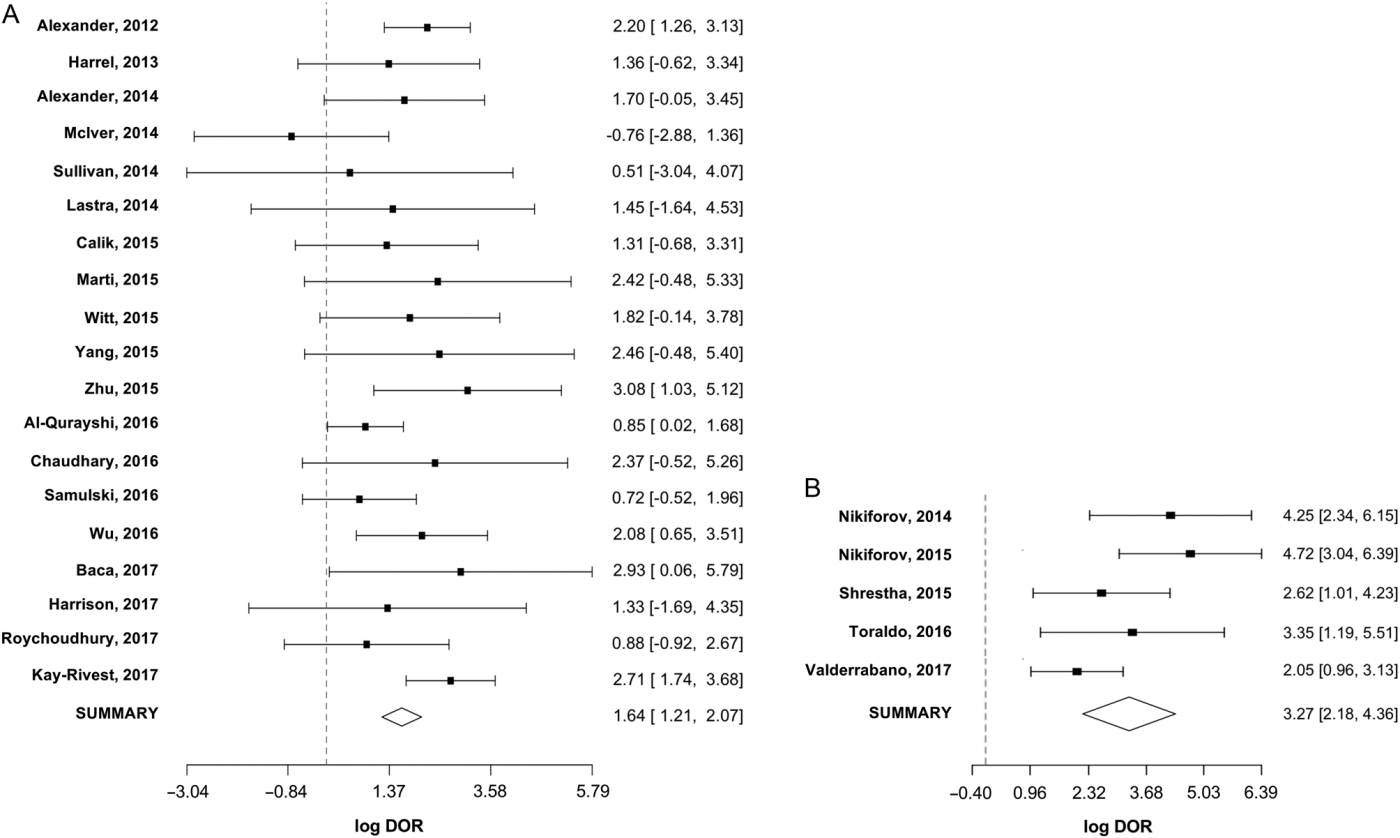
(A) Log diagnostic odds ratio (log-DOR) for the 19 studies of Afirma-GEC. For each one, the dot represents the absolute value of log-DOR, and lines represent the 95% of confidence interval. The values of log-DOR (with 95% of confidence interval) appear at the right of the figure. The diamond represents the summary log-DOR. Dashed lines indicate the line of null effect. B. log-DOR for the 5 studies of ThyroSeq v2.

**Figure 6:**
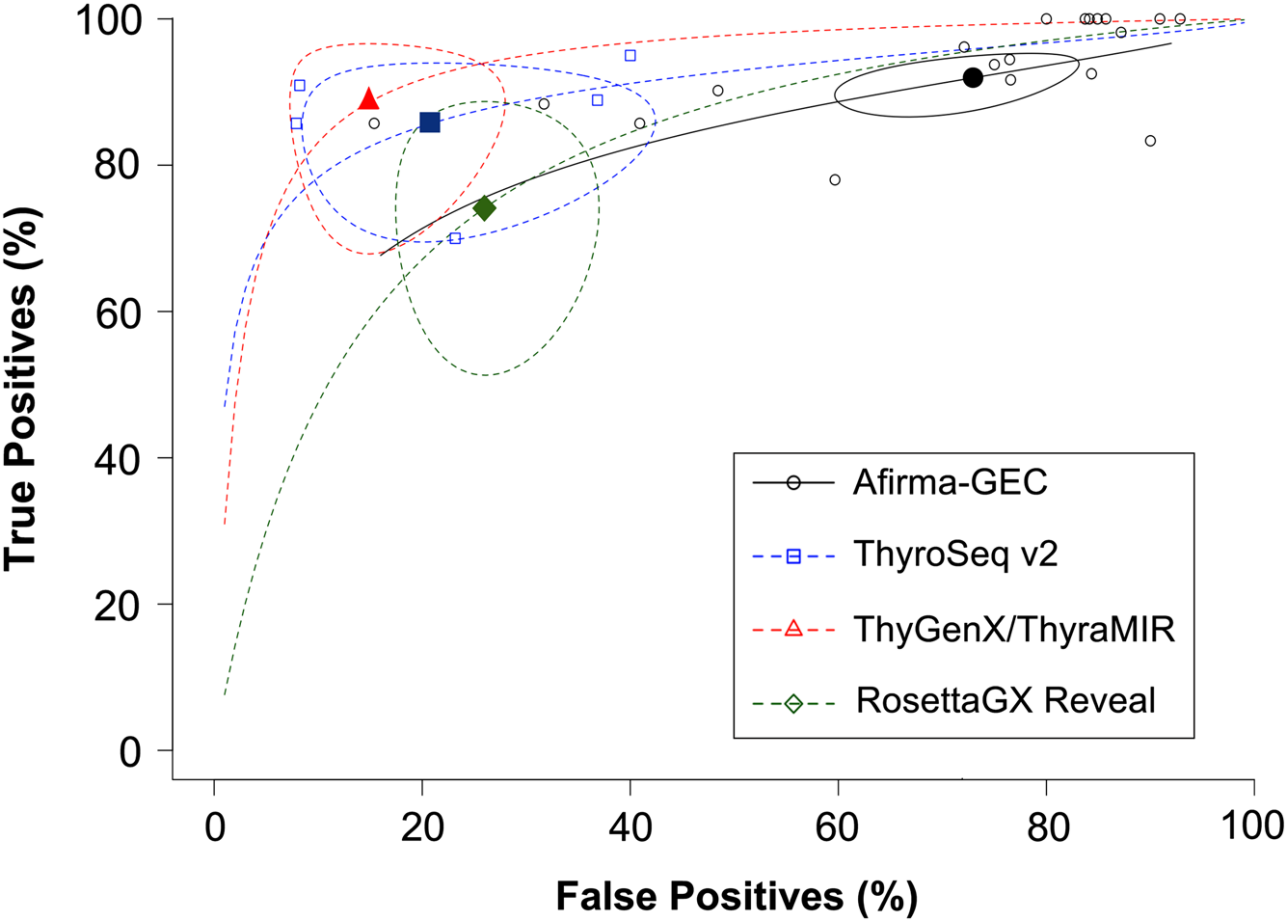
Hierarchical summary receiving operator characteristic (HSROC) curves according the evidence available for the four tests: Afirma-GEC (black), ThyroSeq v2 (blue), ThyGenX/ThyraMIR (red) and RosettaGX Reveal (green). The model represents the relationship of true positives (*y*-axis) at different false positive proportions (*x*-axis), based on the Rutter-Gatsonis model. Empty dots represent single post-validation studies, while filled dots are the summary false positive/true positive pairs after the meta-analysis. Ellipsoids represent the 95% of confidence region.

Meta-regression was performed for Afirma-GEC and ThyroSeq v2, since post-validation studies were available only for these tests. The *I* ^2^ statistic for sensitivity and specificity was very low in both, Afirma-GEC and ThyroSeq v2. Studies based on the Afirma-GEC showed heterogeneity of 9% for sensitivity and 13% for specificity, while the five studies for ThyroSeq v2 showed an *I* ^2^ of 7% for sensitivity and 2% for specificity. In 19 studies analyzed for Afirma-GEC, 14 showed sensitivity over 85%. Four of the five remaining studies had a sample size of 36 patients or less (Fig. 4A). On the other hand, specificity was over 50% in four studies, including the clinical validation study (Fig. 4A). The summary log-DOR was 1.64 (95% CI 1.21–2.07), showing a net benefit for using Afirma-GEC in the diagnosis of ITC (Fig. 5A). For ThyroSeq v2, the three post-validation studies showed sensitivities from 60% to 80% (Fig. 4B). In two post-validation studies, the specificity was near 90%, while in the third study, it was 70% (Fig. 4B). The log-DOR significantly favored the use of ThyroSeq v2 in all the studies, where the summary log-DOR was 3.27 (95% CI 2.18–4.36) (Fig. 5B). A hierarchical summary ROC was modeled based on the accumulated data obtained from all the studies selected, as shown in Fig. 6. For Afirma-GEC, the summarized sensitivity and specificity were 92% and 27%, respectively, while for ThyroSeq v2, those were 86% and 79%. The summarized area under the curve was 0.81 for Afirma-GEC and 0.89 for ThyroSeq v2. Given these parameters, and considering a cancer prevalence of 25% in ITC, the expected NPV for Afirma-GEC was 91% and 94% for ThyroSeq v2. Likewise, the expected PPV for Afirma-GEC and ThyroSeq v2 was 30% and 58%, respectively (Table 4). The meta-regression demonstrates no differences between the NPV of the four tests, and two tests showed a PPV greater than 50% (ThyroSeq v2 and ThyGenX/ThyraMIR). Further details about the paired analysis by meta-regression are shown in Table 4.

**Table 4:** Meta-regression and paired AUC, sensitivity, specificity, likelihood ratios and predictive values (on a cancer prevalence of 25%).

Test	AUC	Sensitivity (%)	Specificity (%)	Negative likelihood ratio	Positive likelihood ratio	Negative predictive value (%)	Positive predictive value (%)
Afirma-GEC	0.81	92	27	0.30	1.26	91	30
ThyroSeq v2	0.89	86	79	0.18	4.10	94	58
ThyGenX/ThyraMIR	0.93	89	85	0.13	5.93	96	66
RosettaGX Reveal	0.80	74	74	0.35	2.85	90	49

AUC, area under the receiving operator characteristic curve.

## Summary of commercially available tests

Considering all the available evidence, we present a summary of the main characteristics of each molecular test for diagnosis of ITC (Table 5). Afirma-GEC has the largest accumulated evidence. Consistent sensitivity (~90%) has been reported when comparing the post-validation studies with the clinical validation. However, in post-validation studies, specificity showed differences with respect to that reported in the clinical validation study (52%), where the consolidated specificity was 27%. Regarding the clinical effectiveness, the number of tests needed to correctly diagnose either a benign or malignant nodule is 2.3, while from each 10 tests performed, one cancer case would be missed. Since the cost-effectiveness of the test is directly proportional to the number of surgeries avoided for each one misclassified cancer patients (SAMC), and inversely proportional to the NND and the cost of the test (C$), we calculated a ‘cost-effectiveness factor’ (CEF) as SAMC/(NND × C$), so the greater the CEF, the greater the cost-effectiveness. For Afirma-GEC, the CEF was 0.91, considering a test cost of USD4800 (Nishino 2015). For ThyroSeq v2, a high overall diagnostic accuracy has been replicated in five different studies, three of them in a post-validation stage. The expected proportion of surgeries avoided could be as much as 79%, with a SAMC close to 17. This performance is associated with a NND of 1.2, and a CEF of 4.43 for an estimated cost of the test of USD3200 (Nishino 2015).

**Table 5:** Summary of commercially available tests.

	Afirma-GEC	ThyroSeq v2	ThyGenX/ThyraMIR	RosettaGX Reveal
Diagnostic accuracy	High sensitivity and NPV, but low specificity	High sensitivity, specificity and NPV	High expected sensitivity, specificity and NPV	Limited overall performance
Post-validation studies	Largely validated consistent sensitivity but not specificity	3 post-validation studies consistent sensitivity and specificity	No	No
Potentially avoided surgeries (based on meta-regression and cancer prevalence of 25%)	27%	79%	Theoretically 85%	Theoretically 74%
Surgeries avoided for each 1 misclassified cancer patient	10	17	Theoretically 23	Theoretically 9
Number needed to diagnose	2.3	1.2	Theoretically 1.2	Theoretically 1.4
Availability	Lab developed test in US	Lab developed test in US	Lab developed test in US	Lab developed test in US
Cost (USD)	4800	3200	3300	3000
Cost-effectiveness factor	0.91	4.43	5.81	2.14

Finally, only theoretical estimations were performed for ThyGenX/ThyraMIR and RosettaGX Reveal, since no post-validation studies have been reported. ThyGenX/ThyraMIR could potentially avoid 85% of unnecessary surgeries, with a SAMC near to 23 and NND of 1.2, similar to ThyroSeq v2. Considering an estimated cost of USD3300 (Nishino 2015), the CEF should be 5.81. In the RosettaGX Reveal case, the test performance could allow to potentially avoid 74% of surgeries for benign nodules. The SAMC, NND and CEF theoretically could be around 9, 1.4 and 2.14, respectively, considering an estimated cost of USD3000 (Nishino 2015).

## Conclusions and perspectives

In the last seven years, novel and promising molecular tests to diagnose ITC have emerged. In this systematic review and meta-analysis, we address the basic concepts needed to critically analyze the quality of the accumulated evidence, performing a comprehensive analysis to better interpret the test results and understand their benefits and limitations. We addressed three critical issues about molecular testing in ITC: (i) the proper theoretical accuracy, considering its intended clinical use (rule-in vs rule-out); (ii) the quality of the evidence reported for each test and (iii) how accurate and effective the tests proved to be in clinical practice.

Based on the accuracy and clinical-effectiveness analysis, a sensitivity of 92% and specificity of 80% appear to be ideal for an ITC test to have an appropriate clinical performance in a wide range of disease prevalence. Importantly, the knowledge of institutional disease prevalence in ITC is necessary for clinicians to anticipate the diagnostic performance of a given test in their particular practice.

The post-validation evidence available for Afirma-GEC and ThyroSeq v2 can be qualified as intermediate-to-good quality evidence. Interestingly, although the between-study heterogeneity is very low for both tests, ThyroSeq v2 shows a slightly lower *I* ^2^ statistic than Afirma-GEC. Possibly, the effect of pre-analytical variations (e.g. sample procurement, inter-population genetic background) could be more easily mitigated by tests based on a mutational profile rather than those with an algorithmic integration of gene expression values. This suggest that, to fairly compare different test performances, the post-validation analysis should consider as much studies as necessary to elude this effect, as showed in this review where *I* ^2^ statistic were comparable for the Afirma-GEC and ThyroSeq v2 body of evidence.

A potential limitation of this meta-analysis is that, for post-validation studies, we only considered cases with surgical pathology and, therefore, cases called benign by molecular testing were excluded. There is controversy on how to consider samples classified as benign that did not undergo surgery. Some authors claim that only those benign cases that ultimately underwent surgery (by other surgical indication) should be considered in the final post-validation analysis. However, this analysis could have a selection bias toward malignant cases, changing the cancer pre-test probability and, therefore, the reported predictive values. Alternately, some authors include both, surgical and benign (non-surgical) cases, assuming that molecular-benign cases are truly benign after one year of follow-up. However, since none of the tests have 100% of NPV, some cases could be false negatives, which eventually could grow very slowly. Probably, a large prospective cohort is required to determine how many benign nodules behave as truly benign and therefore were adequately recommended to follow-up.

For patients with ‘malignant’ result, it would be possible to associate different test results with clinical prognostic outcomes, such as recurrence and mortality. Long-term follow-up could also provide strong evidence about the cost-effectiveness of the test, considering the number of tests performed, how many of these tests changed the treatment decision and the net costs-reduction for healthcare systems.

Another significant consideration is how the test results should be reported. Currently, molecular test results are reported as dichotomic, i.e. ‘positive’ or ‘negative’ for malignancy. Nonetheless, we think that the ‘genetic expression profile’ (e.g. Afirma-GEC) or the ‘mutational profile’ (e.g. ThyroSeq v2) could be more informative about the real risk of a patient to have cancer. For this purpose, it would be interesting to develop studies assessing the probability of malignancy at different gene expression score ranges or the probability of malignancy as the gene mutations accumulate, so clinicians could have an improved approximation about how potentially malignant or benign the nodule is. Furthermore, this non-dichotomic approach to report and interpret the results could be helpful to drive a treatment decision, since more abnormal gene expression scores or mutational profiles could be associated with more aggressive tumors. To prove this, further studies should be performed assessing the clinical outcome of patients with different test results.

Finally, post-validation experiences of other tests (ThyGenX/ThyraMIR and RosettaGX Reveal), as well as newly developed tests, should enrich the discussion about which test to consider for a given setting of patients. We hope that this work provides significant and useful information for healthcare system decision-makers to consider the use of molecular testing as a public health need, avoiding unnecessary surgical risks and costs.

## Supplementary Material

Supporting Table 1

## Declaration of interest

Hernan Gonzalez holds shares at GeneproDx. Natalia Mena works at GeneproDx. Marcela Lagos and Marcela Henriquez receive honoraria form GeneproDx.

## Funding

This work was supported by the Biomedical Research Consortium (grant no. 13CTI-21526P2).
