# Supplementary material for: Genetic testing for indeterminate thyroid cytology: review and meta-analysis
Source: Endocr Relat Cancer. 2017 Dec 18;25(3):R163–77. doi: 10.1530/ERC-17-0405 (PMC5799829; doi:10.1530/ERC-17-0405)
Supplement: Supporting Table 1 [file erc-25-R163-t001.pdf]

TEST NAME	TEST X

Article Title	Accuracy Study of Test X
Authors (first 3)	XX, YY, ZZ
Year	2015

MT Stage	YES	NO
Technical Efficacy		X
Anaytical Validity		X
Clinical Validity	X	
Clinical Utility		X
Cost-Efectiveness		X

ADEQUACY OF REPORTING (ADAPTED FROM STARD CHECKLIST)

		Assessment Question	YES	NO	N/A	Commentary
Title		Is the study identified as a diagnosis accuracy one, with at least one meassure of accuracy? (e.g. Sn, Sp, Predictive Values, AUC)		X		
Abstract		Is there a strutured summary of design, methods, results and conclusion?		X		
Introduction		Is there a scientific and clinical background, including intended use and clinical role?	X			
		Are the objectives and hypothesis specified?	X			
Methods	Study Design	Was data collection performed prospectivelly?	X			
	Participants	Are inclusion and exclusion criteria clearly specified?	X			
		How the enrollment was performed?	X			
		Is the cohort formed by a consecutive collection of patients, instead a random or convenience seires?	X			
	Test Methods	Is the index test detailed as much as to allow its replication?	X			
		Is the reference test detailed as much as to allow its replication?		X		
		Is it explained the rationale for choosing the reference standard?		X		
		(in case of non-training cohorts) Is the cut-off value specified for the index test?		X		
		(in case of non-training cohorts) Is the cut-off value specified for the reference test?			X	
		Was the index test result unnown when reference was interpreted?			X	

		Was the reference test result unnown when index was interpreted?	X			
	Analysis	Were the methods used to assess accuracy appropriate?		X		
		Were the "indeterminate results" properly handled?		X		
		Were "missing data" properly handled?		X		
		There was any analysis of variability in diagnostic accuracy? (differences between training v/s validation results)	X			
		Was the sample size correctly calculated by statistical methods?	X			
Results	Participants	Is there a diagram that shows the flow of participants?		X		
		Are the demographic and clinical characteristics properly described?		X		
		Is the severity of disease detailed?		X		
		Is the distribution of alternative diagnoses detailed?		X		
		Is the time interval between index test and reference standard aproppriate?			X	
	Test Results	Is the cross-tabulation (2 x 2 table) specified for diagnostic accuracy?		X		
		Is the precision of diagnostic estimates specified?		X		
		Were any adverse effect of performing the tests reported?	X			
Discussion		Were limitations (e.g. sources of bias, uncerteinty, potential utility) discussed?	X			
		Were implication for clinical practice (i.e. intended use and clinical role) discussed?		X		

**OVERALL ADEQUACY OF REPORTING**	**Low**	**Medium**	**High**
	X		

RISK OF BIAS

		**Bias-Assessment Question**	**YES**	**NO**	**N/A**	**Commentary**
**Population**	**Spectrum Effect**	Was the test performed in a consecutive collected cohort, avoiding Case-Control?	X			
	**Context Bias**	Was the prevalence manteined in a broad range, similar to real prevalence?		X		
	**Selection Bias**	Did the study avoid inappropriate exclusions?	X			
**Test Protocol**	**Test Execution**	Was the test execution protocol described?	X			
	**Test Technology**	Is the used technology and operators experience controlled?	X			
	**Treatment Paradox**	Was the Reference Test performed with unknowing of MT result?	X			
	**Disease-Progression Bias**	Was the Reference Test performed within a proper time from the MT?			X	

**Verification Procedure**	**Innapropriate Reference Standard**	Is the Reference Test likely to correctly classify the target condition?		X		
	**Differential Verification Bias**	Is the Reference Test the same between different studies?	X			
	**Partial Verification Bias**	Are all the samples verified by the Reference Test?	X			
**Interpretation**	**Diagnosis Review Bias**	Is the MT result unnown when Reference Test is interpreted?			X	
	**Test Review Bias**	Is the Reference Test result unknown when MT is interpreted?	X			
	**Clinical Review Bias**	Is any patients clinical data known when MT nor Reference results is interpreted?		X		
	**Incorporation Bias**	Is the MT result avoided to be used as the final diagnostic?		X		
	**Observer Variability**	Is the test result simmilarly interpreted by different observers?	X			
**Analysis**	**Indeterminate Result**	Are uninterpretable results include in the final analysis?		X		
	**Threshold Value**	Is the threshold value the same between different cohorts?		X		

**OVERALL RISK OF BIAS**	**Low Risk**	**Medium Risk**	**High Risk**
		X	

SAMPLE SIZE

**Assessment Question**	**YES**	**NO**	**N/A**	**Commentary**
Is the Sample Size correctly calculated by statistical methods?	X			

DATA ANALYSIS

**Assessment Question**	**YES**	**NO**	**N/A**	**Commentary**
Is the Data Analysis performed by proper statistical methods?		X		

ETHICS

**Assessment Question**	**YES**	**NO**	**N/A**	**Commentary**
Are all the ethics issues properly addressed?	X			

APPLICABILITY TO THE BODY OF EVIDENCE

		**Assess**	**YES**	**NO**	**N/A**	**Commentary**
**PICOTS**	**Population**	justification for lumping/split + identification/selection method + inclusion/exclusion criteria + demographic characteristics + pre-test probability + spectrum of disease	X			

	**Intervention**	test version + how the test is conducted in practice + who conduct the test in practice + cut-off/thresholds + skills required to interpret the results	X			
	**Comparator**	using of a Gold Standard + alternate tests + management strategy to use the test (replacement, add-on or triage) + comparison with no-testing or usual MT	X			
	**Outcome - Testing Process**	accuracy (Sn, Sp, LR´s, NPV and PPV, AUC, Diagnostic OR, Accuracy and Error)	X			
	**Outcome - Test Result**	clinical outcomes, e.g. earlier diagnosis, earlier intervention, change in care sequence/intensity, change in testing sequence, QALY, costs	X			
	**Timing**	time to make results available for care team + placement of MT in sequence of care + timing of follow-up	X			
	**Setting**	setting of cares (primary v/s speciality) + in-patient v/s out-patient + lab requirements (e.g. centralized, Point of Care, trained personnel) + MT strategy (screening, diagnostic or prognostic)	X			
**Contextual Factors**	**Spectrum Factors**	Is there a possible effect of any of these contextual factors?		X		
	**Management Strategy**			X		
	**MT Methods**			X		
	**Secular Trends**			X		
	**Preventive Care**			X		
	**Treatment Trends**			X		
**OVERALL APPLICABILITY**		**Low**	**Medium**	**High**		
				X		
